# Malaria Death in an Isolated Island Garrison on New Guinea 1915

**DOI:** 10.3390/tropicalmed1010002

**Published:** 2016-07-13

**Authors:** G. Dennis Shanks

**Affiliations:** 1 Australian Army Malaria Institute, 4051 Enoggera, Australia; dennis.shanks@defence.gov.au; Tel.: +61-7-3332-4921; Fax: +61-7-3332-4800; 2 School of Population Health, University of Queensland, 4051 Brisbane, Australia

If I should die, think only this of me:

That there’s some corner of a foreign field

That is forever England.

Rupert Brooke, 1914

Most military cemeteries overwhelm one with the vast number of markers that represent once living soldiers now buried far from home. Occasionally one finds a lonely, single grave of an unremembered death and wonders what must have happened many years ago. Most people will know of the famous poet Rupert Brooke who died of bacterial sepsis just prior to the Gallipoli landings, who is buried on the Greek island of Skyros and whose quotation appears above. However, not long before Gallipoli, another amphibious operation had taken place on the other side of the world and both the landing and its casualties are now largely forgotten. During antimalarial drug testing on Bougainville sixteen years ago, the staff of the Australian Army Malaria Institute came upon a lonely grave in Keita and its photograph appears as Figure 1. The extensive on-line databases kept by the Australian War Memorial, the Australian National Archives, and the Commonwealth War Graves Commission allows us to discover what happened 100 years ago as a means of respecting an individual sacrifice as well as appreciating just how much tropical medicine has changed in the last century [1,2,3].

The Australian Naval and Military Expeditionary Force (ANMEF) was one of the first military actions of the First World War, deploying from Sydney in August 1914. The ANMEF was a rapidly raised, independent Australian force consisting of a mixed contingent of 2000 men sent north to capture the German colony of New Guinea [4]. After stopping in Port Moresby to pick up Queensland reinforcements, it arrived at Rabaul on 11 September 1914. The subsequent landings were opposed by German reservists and Melanesian police leading to seven Australian deaths (including one Royal Australian Army Medical Corps (RAAMC) officer) prior to the occupation of the area, capture of the radio station, and the ending of hostilities the following day. The apparent accidental loss of the submarine AE1 on 14 September added 35 further deaths to the casualty list. Since only tropical garrison duties were anticipated after the destruction of the German fleet off the Falkland Islands on 8 December 1914, most of the ANMEF left at the end of their six month enlistment to join the Australian Imperial Force then forming in the Egyptian desert prior to the ill-fated Gallipoli campaign.

The several hundred men of the residual Australian forces in New Guinea then had to establish a civil-military administration across many islands with little infrastructure other than scattered coastal plantations. Small detachments were sent to the outlying areas to keep the appearance of government functioning including Madang on the northern coast, Lorengau on Manus, Angorum up the Sepik River, and Kieta on Bougainville. Usually these isolated outposts consisted of one to three officers (one being a medical officer), 20 other ranks, and some local policemen initially with no radio capability or dedicated boats for transport [1]. Bougainville was particularly important as the presumed cannibal tribes from the island’s interior were threatening the plantation owners on the coast. The first ANMEF detachment to Kieta, including PTE Read, arrived prior to Christmas 1914.

In January 1915 a long drought was broken and the subsequent rains initiated a malaria epidemic which infected most of the Rabaul garrison and filled the hospital. The outpost at Angorum was abandoned following malaria deaths of two soldiers. Daily compulsory quinine administration (Figure 2) for parasite suppression was thoroughly disliked by the men due to the foul taste of the liquid preparation used. It is likely that PTE Read was infected in January and he is known to have died 11 February 1915. He is one of eight known malaria deaths to have occurred in the ANMEF [1]. The stated diagnosis of blackwater fever (see Figure 3) usually requires months of exposure with chronic, intermittent quinine use eventually leading to a massive hemolytic reaction of unknown pathogenesis. Blackwater fever was a greatly feared aspect of tropical life during the colonial era and a major cause of death in expatriate populations, who often regarded it as untreatable. Unless PTE Read had been accustomed to using chronic quinine from his experience during the Boer War in South Africa, it is unlikely the lethal infection he developed could be considered classical blackwater fever as it happened too soon after his arrival in the tropics. An alternative view of the final illness would be that of an overwhelming falciparum infection which led to hyperparasitemia with associated hemolysis and renal failure causing death. Such a fulminating *P. falciparum* infection killed the last known Australian soldier to die of malaria in December 1965 in South Vietnam, mid-way in time between PTE Read’s death and the present [5]. *P. falciparum* remains one of the few infectious diseases capable of rapidly killing an otherwise healthy adult and requires a high level of awareness following visits to endemic areas, as recently demonstrated during a successfully treated malaria outbreak on the HMAS Newcastle while on patrol in the Indian Ocean.

Shortly after the end of the war, the Australian War Memorial’s Roll of Honour was formed and postal inquiries were sent to family members to obtain further details of those who had died. PTE Joseph Read was a 50-year-old plasterer living in South Australia, originally from England having immigrated to Australia in 1912. PTE Read would have been one of the few experienced soldiers in the ANMEF, having served in the Second Battalion of the Dorsetshire (39th) Regiment during the South African War in 1900–1901, and he had been awarded the Queen’s South Africa medal with five bars. There was no mention of surviving family members other than a brother in England.

PTE Read’s grave is a solitary one being the only Commonwealth War Graves Commission burial in the Kieta cemetery. Other ANMEF malaria deaths occurred in Madang and Lae. Disease deaths seem to be often discounted against the apparently more noble image of death by missile injury in the face of the enemy. There were all too many of such traumatic deaths to follow during the First World War, as the massive cemetery at Tyne Cot, France with 11,956 burials and 34,946 memorials for those with no known grave, attests. As medical persons interested in tropical medicine we should strive, particularly during the events commemorating the centenary of the First World War, to remember those whose deaths were caused by infectious diseases and not let their equal sacrifice go un-noticed. Our struggle against infectious diseases is far from over and Bougainville remains one of the most malarious islands of the Pacific.

## Figures and Tables

**Figure 1:**
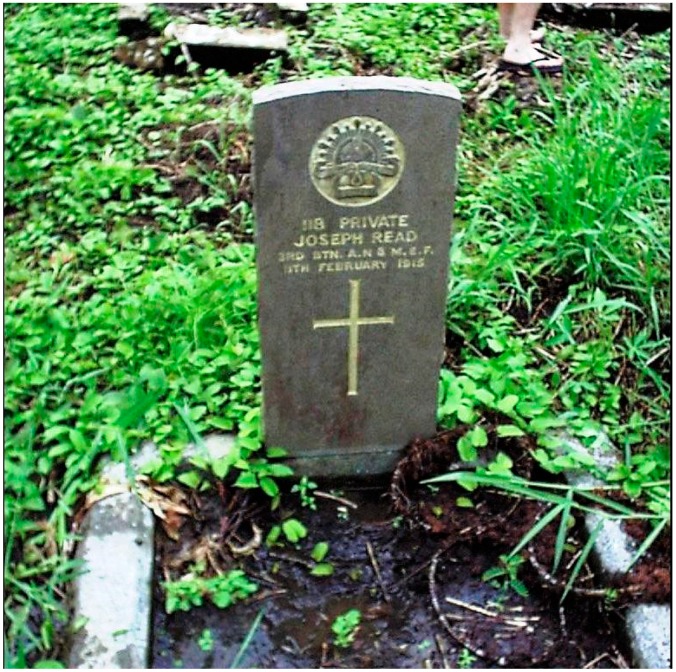
Commonwealth War Graves Commission tombstone marking the grave of 188 PTE Joseph Read of the Australian Naval and Military Expeditionary Force to New Guinea, who died in Kieta, Bougainville on 11 February 1915 from blackwater fever.

**Figure 2:**
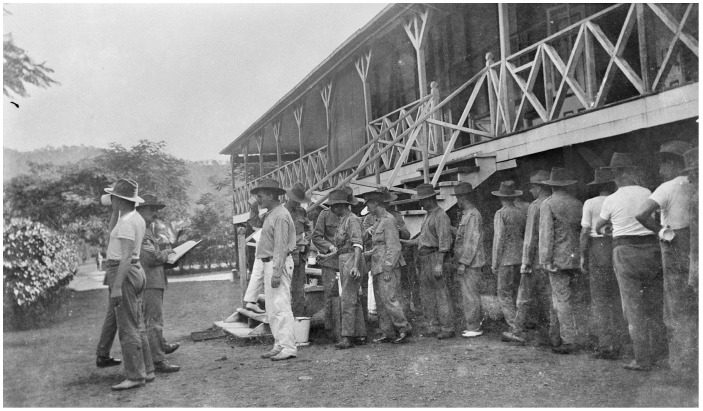
Daily directly-observed administration of quinine to the Rabaul garrison of the Australian Naval and Military Expeditionary Force was used to suppress the near universal *Plasmodium* infection of the soldiers. Australian War Memorial photo J02924.

**Figure 3:**
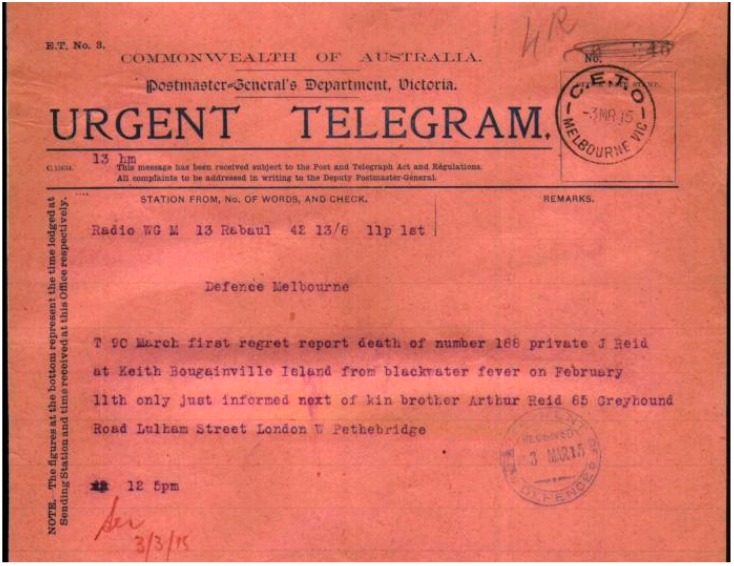
Radio telegram from Rabaul, New Guinea in March 1915 informing headquarters of the death of PTE Read (Reid) and that his brother as next of kin in London had been informed of the death. From Australian National Archives online database [2].
